# Class effect of pharmacotherapy in bipolar disorder: fact or misbelief?

**DOI:** 10.1186/1744-859X-10-8

**Published:** 2011-03-24

**Authors:** Konstantinos N Fountoulakis, Xenia Gonda, Eduard Vieta, Zoltan Rihmer

**Affiliations:** 1 Third Department of Psychiatry, School of Medicine, Aristotle University of Thessaloniki, Greece; 2 Department of Pharmacodynamics, Semmelweis University, Budapest, Hungary; 3 Department of Clinical and Theoretical Mental Health, Faculty of Medicine, Semmelweis University, Budapest, Hungary; 4 Bipolar Disorders Program, Hospital Clinic, IDIBAPS, CIBERSAM, University of Barcelona, Barcelona, Spain

## Abstract

**Background:**

Anecdotal reports suggests that most clinicians treat medications as belonging to a class with regard to all therapeutic indications; this means that the whole 'class' of drugs is considered to possesses a specific therapeutic action. The present article explores the possible existence of a true 'class effect' for agents available for the treatment of bipolar disorder.

**Methods:**

We reviewed the available treatment data from randomized controlled trials (RCTs) and explored 16 'agent class'/'treatment issue' cases for bipolar disorder. Four classes of agents were examined: first-generation antipsychotics (FGAs), second-generation antipsychotics (SGAs), antiepileptics and antidepressants, with respect to their efficacy on four treatment issues of bipolar disorder (BD) (acute mania, acute bipolar depression, maintenance against mania, maintenance against depression).

**Results:**

From the 16 'agent class'/' treatment issue' cases, only 3 possible class effects were detected, and they all concerned acute mania and antipsychotics. Four effect cases have not been adequately studied (FGAs against acute bipolar depression and in maintenance protection from depression, and antidepressants against acute mania and protection from mania) and they all concern treatment cases with a high risk of switching to the opposite pole, thus research in these areas is poor. There is no 'class effect' at all concerning antiepileptics.

**Conclusions:**

The available data suggest that a 'class effect' is the exception rather than the rule in the treatment of BD. However, the possible presence of a 'class effect' concept discourages clinicians from continued scientific training and reading. Focused educational intervention might be necessary to change this attitude.

## Background

In the last decade there were important developments in our understanding of bipolar disorder (BD), as well as its treatment. From a historical point of view, since Hippocrates from antiquity to Emil Kraepelin in the early 20th century, manic depressive illness has been established as a nosological entity (and separate from schizophrenia) on the basis of heredity, longitudinal follow-up and a supposed favorable outcome. However, recently there was important insight into the illness with the description and definition of subtypes (BD-I to BD-VI) [1-3].

This dramatically changed the perceived epidemiology of the disorder. Although earlier studies suggested that the classic manic depressive psychosis had a prevalence of around 1% (0.4% to 1.6%), today we know that the true prevalence depends on the definition, and with the inclusion of subthreshold bipolar cases, pseudounipolar patients and personality disorders (PDs), especially 'borderline personality disorder' under the umbrella of the 'bipolar spectrum', the combined prevalence rate is up to 3.7%, with BD-II being the most prevalent subtype [4-6].

Similarly, our knowledge and understanding of treatment has progressed, largely following our understanding of the clinical picture. The first dramatic conclusion was that the outcome of bipolar illness is not favorable as Kraepelin had determined, but rather suboptimal and is strongly related to younger age of onset and to alcohol and substance abuse. Following this fact, the World Health Organization (WHO) has recently ranked bipolar disorder among the 10 most disabling medical conditions worldwide [7].

Today we know that the treatment of bipolar illness is complex and full of caveats for the clinician [8-11], with some aspects of the disorder being rather refractory to treatment. One widespread concept among clinicians is that of the so-called 'class effect'. As most pharmaceutical agents belong to a 'class' usually on the basis of their primary therapeutic labeling (for example, antipsychotics, anticonvulsants, antidepressants and so on), it seems that clinicians use them according to the 'class' they belong rather than on the basis of the individual substance and its properties. This means that the whole 'class' of drugs is considered to possesses a specific therapeutic action that in some cases has been proven only for a few of its members or even only for a single one. An example in the case of BD could be that all 'antiepileptics' are also 'mood stabilizers'. Clinicians and researchers seem to treat agents in a 'class' way, by, for example, suggesting that antidepressants do not work in bipolar depression in spite of the fact that negative data exist only for a few of them while positive data might exist for fluoxetine.

If such a situation exists, and patients usually receive treatment according to a 'class effect', it has huge implications for public health and also for the overall cost of mental disorders, and especially for bipolar disorder. The 'class effect' provides the clinician with fast and simple rules to determine treatment, but in the case of bipolar disorder it might provide the clinician with oversimplified and false rules, and might result in a significant proportion of patients receiving the wrong treatment.

The present work aims to determine whether such 'class effects' are present in bipolar disorder. On the basis of available evidence, several reviews and meta-analysis papers [12-16] developed tables concerning the efficacy of various agents in the different faces and facets of bipolar illness (Table 1). The question was whether these tabulated data support or call into question the presence of a 'class effect' for the treatment of bipolar disorder.

**Table 1:** Monotherapy data on the efficacy of agents and classes of agents in different phases of bipolar illness

Agent/modality (alphabetical order)	Acute mania	Acute bipolar depression	Maintenance against mania	Maintenance against depression
FGAs	Class effect	Unknown	Uncertain class effect	Unknown

Chlorpromazine	Positive	-	-	-

Haloperidol	Positive	-	-	-

Perphenazine	-	-	Negative	-

SGAs	Class effect	No class effect	Class effect	No class effect

Amisulpride	-	-	-	-

Aripiprazole	Positive	Negative	Positive	Negative

Asenapine	Positive	-	-	-

Clozapine	Positive	-	-	-

Olanzapine	Positive	Equivocal	Positive	Positive

Olanzapine-fluoxetine combination	-	Positive	-	-

Paliperidone	Positive	-	-	-

Quetiapine	Positive	Positive	Positive	Positive

Risperidone	Positive	-	Positive	-

Ziprasidone	Positive	-	Positive	-

Antiepileptics	No class effect	No class effect	No class effect	No class effect

Carbamazepine	Positive	Equivocal	Equivocal	Equivocal

Gabapentin	Negative	-	-	-

Lamotrigine	Negative	Negative	Negative	Positive

Licarbazepine	Negative	-	-	-

Topiramate	Negative	-	-	-

Valproate	Positive	Equivocal	Equivocal	Equivocal

Antidepressants	Unknown	No class effect	Unknown	No class effect

Fluoxetine	-	Positive	-	Positive

Paroxetine	-	Negative	-	-

Venlafaxine	-	Equivocal	-	-

- = No data available.

## Methods

We reviewed the available treatment data from randomized controlled trials (RCTs). These treatment datasets have already been published previously [12-16], so only minor additions were necessary. Four classes of agents were examined: first-generation antipsychotics (FGAs), second-generation antipsychotics (SGAs), antiepileptics and antidepressants, with respect to their efficacy on four treatment issues of BD (acute mania, acute bipolar depression, maintenance against mania, maintenance against depression). This led to a 4 × 4 crosstabulation with 16 'agent class'/'treatment issue' cases.

Although such tables already exist in previous works, we created a new one on the basis of a fresh look at the available data. In spite of a 'general acceptance' of treatment options for bipolar disorder, the evidence shows a much different picture. Thus, a review of the literature was judged to be necessary. If the opposite were the, case the table would rely on arbitrary opinion and could be misleading.

## Results

### Effective treatments for bipolar disorder

Valproate has proven efficacy against acute mania [17-22]. There are only two small positive studies suggesting it might be effective in reducing the symptoms of depression and anxiety in bipolar I patients during the acute depressed episode [23,24], (two more on the extended release form of valproate, one positive and one negative, have not been published [25,26]). One maintenance phase RCT was negative for valproate, however, it possibly suffered from a problematic study sample [27].

Carbamazepine is efficacious against acute mania [28-31], but with regard to acute bipolar depression there is only one dated positive small withdrawal study [28] and this is also the case for maintenance [32].

Lamotrigine is not effective against acute mania (two unpublished negative RCTs exist; SCAA2008 and SCAA2009) [16] and its efficacy in acute bipolar depression is controversial (five RCTs were negative on the primary outcome; SCA100223, SCA30924, SCA40910, SCAA2010 and SCAB2001 [33]; however, one of those was clearly positive on the Montgomery-Åsberg Depression Rating Scale (MADRS) [34] and the only adjunctive RCT was positive when lamotrigine was combined with lithium [35]. In contrast, there is strong evidence that during the maintenance phase, lamotrigine protects from depressive episodes but not from mania [36-40].

The data concerning the acute manic phase are negative for gabapentin [41] and topiramate [42].

There are some data concerning the efficacy of FGAs against acute mania but there are no data against bipolar depression or the maintenance phase. There is only one early, small, placebo-controlled study supporting the efficacy of chlorpromazine [43]. Several studies support the efficacy of haloperidol [44-48]. Most clinicians and experts believe that typical antipsychotics induce the opposite pole and cause dysphoria and depression. However, this has only been reported concerning haloperidol and perphenazine, suggesting that they could decrease the time to switch into depression compared with atypical antipsychotics [49,50].

There are data supporting the efficacy of most SGAs against acute mania. However, data against acute bipolar depression and concerning maintenance are not homogenous.

Olanzapine has proven efficacy against acute mania [51-55]. Although there are also positive data concerning acute bipolar depression [56] there is concern on the effect on the 'depressive core' of symptoms although it is certain that the patients manifested a significant improvement in symptoms 'peripheral' to the definition of depression such as insomnia, anxiety, loss of appetite and so on [57,58]. Maintenance data are positive concerning protection from manic, depressive and mixed episodes with olanzapine [59,60] and with olanzapine-fluoxetine combination (OFC) [56,61-64].

Quetiapine has proven efficacy against acute mania [45,65,66], including an unpublished study (NCT00309699). The data are solid also against acute bipolar depression [67-74] and they are also effective against depression in bipolar II depression [75]. Combination data with a mood stabilizer and monotherapy are available concerning the maintenance phase [76,77].

Aripiprazole is efficacious against acute mania [78-80] although one RCT with a fixed dosage design was negative [81]. Data are negative concerning bipolar depression [82]. During the maintenance phase it is reported to protect from manic relapses but not from depressive relapses [83,84]. Risperidone is efficacious against acute mania [46,85-87]. Recently, positive data concerning the maintenance phase became available for long-acting injectable risperidone, suggesting it is effective in the prevention of manic or mixed episodes but not depressive episodes [88]. Ziprasidone is efficacious against acute mania [48,89-91]. Data are negative concerning bipolar depression (two unpublished studies). There is one positive maintenance RCT with ziprasidone as an adjunct to valproate or lithium [92]. Asenapine is efficacious against acute mania [54,93]. No data are available concerning bipolar depression or the maintenance phase. The data are also positive for paliperidone against acute mania (one positive RCT with flexible dosage (NCT00309699) [94] and one negative with fixed dosage (NCT00299715)), but no data are available concerning bipolar depression or the maintenance phase.

The use and usefulness of antidepressants in bipolar disorder is controversial because of the risk of inducing the opposite pole. By definition, antidepressants are not used against acute mania (and there are no trials during the acute manic phase). From RCTs against acute bipolar depression, older studies suggested that amitriptiline [95] and maybe imipramine could be effective [96-98], with data being somewhat stronger for fluoxetine (particularly in bipolar II patients) [99-102]. As mentioned above, data are strong only for OFC [56,62-64]. At the same time, the data are negative for paroxetine monotherapy [103] and equivocal for venlafaxine, possibly because of a high switch rate [104]. A recent large, naturalistic study showed that up to 15% of the patients with mania receive antidepressants combined with antimanic agents, but this practice was actually associated with poorer outcomes compared to those who did not receive antidepressants [105].

Fluoxetine was reported to be effective during the maintenance phase for bipolar II patients [100,101,106].

The data concerning combination and add-on treatment suggest that in acutely manic patients who are partial responders to lithium, valproate or carbamazepine, a good strategy would be to add haloperidol, risperidone, olanzapine, quetiapine or aripiprazole. Adding oxcarbazepine to lithium could also be a choice [107-124]. Combination data are negative for paliperidone (NCT00309686), positive for asenapine (NCT00145470 and NCT00145509) and negative for licarbazepine. Combination treatment studies in bipolar depression are equivocal [35,96-98,103,115,125-131]. A recent unpublished add-on study with ziprasidone (NCT00483548) was negative. Combination treatment during the maintenance phase includes quetiapine plus a mood stabilizer [76,77]; a discontinuation study on olanzapine as added on lithium or valproate was positive for olanzapine [132], and another discontinuation RCT of the combination of mood stabilizer plus ziprasidone was positive for ziprasidone [92]. Valproate was more effective than lithium when added on antidepressants for the prevention of bipolar depression [133].

A 40-week placebo controlled study of the safety and efficacy of asenapine when added to lithium or valproate (NCT00145509) and a 40-week extension study of asenapine vs olanzapine (Ares 7501007) have also been conducted.

Generally, add-on studies suggest that at least some strategies could be useful in patients with inadequate response to monotherapy. However the recently published BALANCE study could neither reliably confirm nor refute a benefit of combination therapy compared with lithium monotherapy [134] at least partially because of methodological flaws [135]. Overall, there is no compelling data that combination treatment does better than monotherapy. However, patients stabilized on combination treatment might do worse if shifted to monotherapy, and patients refractory to monotherapy could benefit with add-on treatment with olanzapine, aripiprazole, risperidone, quetiapine, ziprasidone, valproate, an antidepressant or lamotrigine, usually depending on the index acute phase.

A summary of the efficacy of various agents against the different phases of bipolar illness is shown in Table 1.

## Discussion

In the current study, from the 16 'agent class'/' treatment issue' cases, only 3 possible class effects were detected. They all concern acute mania and antipsychotics (FGAs and SGAs against acute mania and SGAs in maintenance protecting from mania). Four effect cases are not adequately studied (FGAs against acute bipolar depression and in maintenance protecting from depression, antidepressants against acute mania and protecting from mania) and they all concern treatment cases with high risk of switching to the opposite pole; thus, research in these areas is poor.

What is impressive is the lack of any class effect concerning antiepileptics. This has been reported previously [136], and it is very interesting because anecdotal reports suggest that the average clinician considers the term 'antiepileptic' to be more or less interchangeable with 'mood stabilizer'. However, the data are only positive concerning valproate, carbamazepine and lamotrigine, and only against specific phases of the illness.

From a clinical point of view, depression and the maintenance phase seem to be more important since effective treatments are much fewer in comparison to acute mania. In particular, for the prevention of bipolar depressive episodes, the options seem to be quite limited, and no class effect is present.

Pharmacoepidemiological data are limited and usually concern established treatments such as lithium, valproate or antipsychotics, but they rarely concern non-established treatments such as newer antiepileptics. The lack of this kind of data is especially problematic for bipolar depression. An unpublished poster presentation from Japan reported that Japanese psychiatrists were divided between antidepressants and 'mood stabilizers' on the treatment of bipolar depression [137]. A study from the 1990s utilized the pharmacy records of McLean Hospital from 1987 to 1993 and reported that 3,829 bipolar depressive inpatients had received tricyclic antidepressants, 2,981 fluoxetine, 2,603 trazodone, 809 bupropion, 743 monoamine oxidase inhibitors, 592 stimulants, 588 sertraline, 48 paroxetine, and 894 electroconvulsive therapy [138]. Reports on real-world maintenance treatment suggest a variable picture. Baseline treatment data for the first 500 patients in the Systematic Treatment Enhancement Program for Bipolar Disorder (STEP-BD) study (1998 to 1999) revealed that while standard mood stabilizers (lithium, valproate, or carbamazepine) were the most commonly prescribed class of drugs for participants (71.9%), the use of novel anticonvulsants was high (31.8%) and more frequent than that of SGAs (27.2%) [139]. Antidepressants are also prescribed as if there is a 'class effect' present during the maintenance phase. The US data from non-hospitalized subjects with bipolar I disorder in 1995/1996 suggested that more than half of all subjects were receiving concomitant antidepressants, of whom nearly 50% received selective serotonin reuptake inhibitor (SSRI) antidepressants and nearly 25% received buproprion [140]. The data from the 2002 to 2003 US national MarketScan research databases data suggest that the most commonly prescribed first drug class was antidepressants (50% of patients) [141]. Baseline treatment data for the first 500 patients in the STEP-BD study (1998 to 1999) revealed that the second most common class of agents was antidepressants (40.6%) [139]. In The Netherlands, the search of prescription patterns during 1996 to 2005 revealed a significant decrease in the use of tricyclic antidepressants, which, however, were still in wide use [142]. A Hungarian study reported that 35% of patients were on antidepressants and more than half of them on SSRIs, which implies a sustained wide use of tricyclics [143]. UK data from the case note review of patients from north-east England suggested that 23% of patients were on antidepressants; 11% of them were not prescribed a mood stabilizer and 43% of antidepressants prescribed were tricyclics [144]. Taking the above together, it seems that depending on the sample, 25% to 50% of bipolar patients are cross-sectionally under antidepressants, with almost half of them receiving tricyclics.

It is true that the earlier studies tended to suggest a high and global effectiveness for older agents on all facets of bipolar disorder and a high prevalence of switching with antidepressants. Neither conclusion is confirmed by newer studies; however, since these conclusions were widely accepted for decades, these old agents are considered to be the 'gold standard' and 'class effects' were suggested to exist. The extent to which these 'class effects' truly influence everyday clinical practice worldwide is unknown; similarly the extent they influence the outcome of the treatment and the natural history of the disease is also unknown.

While evidence-based medicine has seemed to dominate medical scientific thinking in the last few decades, this is not true for wider clinical practice. The evidence is limited and hard to interpret and to carry over into everyday practice. However, it is highly likely that a significant number of patients worldwide are not receiving proper treatment simply because the 'class effect' idea discourages continued scientific training and reading. Focused educational intervention may be necessary to change this attitude.

## Conclusions

In the treatment of bipolar disorder, a pharmaceutical class effect is the exception rather than the rule, and such class effects concern only acute mania and antipsychotics. Some facets of bipolar disorder have not been adequately studied to date; however, this does not seem to have influenced the general picture. Since a presumed 'class effect' is a very frequent and not adequately studied factor behind pharmaceutical prescription, the results of the current study suggest that a significant number of patients worldwide may not receive proper treatment. This situation can be corrected only by educational intervention, focused on changing this misconception.

## Competing interests

KNF is/was member of the International Consultation Board of Wyeth for desvenlafaxine, BMS for aripiprazole in bipolar disorder and Servier for agomelatine and has received honoraria for lectures from AstraZeneca, Janssen-Cilag, Eli Lilly and research grants from AstraZeneca and Pfizer Foundation. XG has received travel support from GlaxoSmithKline, Krka, Lilly, Montrose, Organon, Richter, Sanofi, and Schering-Plough. EV has acted as consultant, received grants, or received honoraria for lectures by the following companies: Almirall, AstraZeneca, Bial, Bristol-Myers-Squibb, Eli Lilly, Forest Research Institute, GlaxoSmithKline, Janssen-Cilag, Jazz Lundbeck, Merck-Sharpe-Dohme, Novartis, Organon, Otsuka, Pfizer, Sanofi, Servier, Schering-Plough, Takeda, UBC, and Wyeth. ZR has received speaker's honoraria from AstraZeneca, GlaxoSmithKline, Lilly, Lundbeck, Organon, Pfizer, Richter, Sanofi-Aventis, Servier-EGIS, and Wyeth Pharmaceuticals. He also received honoraria as a member of scientific advisory boards of AstraZeneca, Lilly, Organon, Pfizer, Richer, Sanofi-Aventis and Servier-EGIS.

## Authors' contributions

KNF conducted the literature search, interpreted the results and wrote the first draft, and commented on following drafts. XG contributed to the interpretation of results, wrote subsequent drafts. EV contributed to the literature search and to the interpretation of results and commented on following drafts. ZR contributed to the interpretation of results and commented on following drafts.
